# Brain Metastases from Colorectal Cancer: Microenvironment and Molecular Mechanisms

**DOI:** 10.3390/ijms131215784

**Published:** 2012-11-26

**Authors:** Yi-Wen Zang, Xiao-Dong Gu, Jian-Bin Xiang, Zong-You Chen

**Affiliations:** Department of General Surgery, Huashan Hospital, Fudan University, 12 Middle Wulumiqi Road, Shanghai 200040, China; E-Mails: yiwen.z@163.com (Y.-W.Z.); gxdgxd737@sohu.com (X.-D.G.); xjbzhw@163.com (J.-B.X.)

**Keywords:** colorectal cancer, brain metastasis, microenvironment

## Abstract

Colorectal cancer is one of the most common digestive tract malignancies in the world. Owing to the newer and more effective systemic therapies, the life of colorectal cancer patients can be remarkably prolonged, and the incidence of brain metastases is increasing. However, little is known about the underlying mechanisms of brain metastasis from colorectal cancer. Here we review the tumor microenvironment and metastasis associated molecules in brain metastases from colorectal cancer. A further understanding of these mechanisms will help us to propose better strategies for colorectal cancer patients with brain metastasis and improve their life quality.

## 1. Introduction

Colorectal carcinoma (CRC) is the third most commonly diagnosed cancer in males and the second most common in females worldwide [1]. The main causes of failure for treatments are local recurrence and distant metastases, especially when vital organs such as liver and lung are involved through hematogenous dissemination. Brain metastasis is a much less common but more fatal phenomenon and is usually considered as the late-stage manifestation for CRC. Compared with other malignancies such as lung cancer, breast cancer, and melanoma, the incidence of brain metastasis from CRC is considerably rare. Merely 0.3% to 9% of patients with CRC may develop brain metastasis synchronically or heterochronically [2], representing only 4% to 6% of all brain metastasis cases [3]. Lately, the incidence of brain metastasis has been increasing, owing to the improved radiological techniques and multimodality therapies for systemic diseases, which prolongs the survival rate but at the same time increases the risk for developing brain metastases [4,5]. Considering this, the problem of brain metastasis from CRC should be ignored no longer. Most brain metastases occur in the cerebral hemispheres (48%–58%), followed by the cerebellum (18%–43%), and 23%–33% patients have multiple lesions. Brainstem metastasis of CRC is rare. Patients may complain about headache, motor disturbance, mental change, nausea or vomiting, seizure, aphagia, or visual disturbance according to the functional brain area involved [5–7].

Once diagnosed with brain metastasis, the patient’s prognosis is not promising, with less than 6 months survival expectancy. Several prognostic factors for patients with brain metastasis of colorectal carcinoma have been identified, including age <65 years, shorter interval from CRC diagnosis to BM diagnosis, and >3 intracranial metastases [8,9]. Recent researches concluded that the amount of chemotherapy before brain metastasis [7] were also the independent risk factors for overall survival.

## 2. Molecules Associated with Metastatic Potential

The process of metastatic cascade includes local invasion, intravasation, dissemination, extravasation, and colonization at the new site. As a matter of fact, not all cancer cells in the primary lesion are able to form a metastatic lesion. The primary tumor is usually genetic heterogeneous, consisting of cells with different potentials to migrate and metastasize. Genetic mutations make it possible for some of the cancer cells to gain the ability to metastasize (see in Table 1), yet these malignant cells still need to conquer various environmental pressures (hypoxia, poor blood supply, growth suppressor genes, cell checkpoint, DNA damage system, and immune mediators) to successfully proliferate in the metastatic target organs.

### 2.1. Epidermal Growth Factor Receptor Signaling

Amplification and overdose of epidermal growth factor receptor (EGFR) were identified in several brain metastatic solid tumors, indicating that *EGFR* mutations and amplification are involved in the tumorigenesis of brain metastasis [10]. Scartozzi *et al*. [11] investigated the difference of EGFR statuses between primary CRC and corresponding metastatic sites (liver, lung, bone, and brain). In 19 of 53 primary tumors expressing EGFR, the corresponding metastatic site was found to be negative, whereas it was found to be positive in 15% metastases from EGFR-negative primary cancers. The irrelevance of EGFR status in primary and metastatic CRC can explain to some extent the fact that some CRC patients cannot have a good prognosis after EGFR-targeted monoclonal antibodies, besides through underlying mechanisms the metastatic CRC may alter its biophenotype such as EGFR status to survive from immune elimination and chemotherapeutants.

The mutation of *KRAS*, a member of the rat sarcoma virus gene family of downstream genes of *EGFR*, devotes most part of mutated genes in the EGFR signaling pathway (35%–45% of all CRC), which strongly correlates with poorer outcomes in patients with CRC [12]. Data showed that KRAS mutation prevalence was high in CRC patients presenting with brain metastases compared with primary cancer, and even higher than in liver metastases [13]. These results revealed the importance of KRAS signaling in formation of brain metastasis from CRC.

### 2.2. Metastasis Promoter

#### 2.2.1. CD44

CD44 is an integral membrane glycoprotein that functions as a receptor for the extracellular matrix glycan. The standard isoform, designated CD44s. CD44 splice variants containing variable exons are designated CD44v. CD44 is frequently expressed on primary brain tumors and brain metastases. CD44v expression was weak in primary brain tumors and cell lines derived from normal brain and tumor tissue. However, high levels of isoforms were shown in all metastatic brain tumors [14]. The expression level of CD44s and CD44v6 in CRC were significantly higher in primary tumors as compared to their metastases [15]. Interaction with the CD44-associated signaling by inhibition of fatty acid synthase would reduce metastasis in CRC, which suggests a potential treatment strategy for advanced CRC [16].

#### 2.2.2. Rho GDP Dissociation Inhibitor 2

The potential effect of Rho GDP dissociation inhibitor 2 (RhoGDI2) on cancer cell metastasis was first presented in human bladder cancer cell lines [17]. It functions as GTP-binding proteins of the Ras superfamily and regulates the development of numerous aspects of the malignant phenotype, including cell cycle progression, resistance to apoptotic stimuli, neovascularization, tumor cell motility, invasiveness, and metastasis [18]. Up-regulation of RhoGDI2 would lead to a low activity of Rac and Cdc42 and rector-dependent deficiency in cell migration [19]. Over-expression of wild-type or constitutively active forms of RhoA has been shown to induce invasive behavior in non-invasive rat hepatoma cells *in vitro*. Over-expression of RhoGDI2 in CRC can enhance the cell proliferation, motility, and invasion *in vitro*[20]. RhoGDI2 was an independent prognostic factor for relapse-free survival of CRC in a multivariate analysis [21], and presented as one of the potential multi-drug resistant genes [22].

#### 2.2.3. Smad4

Smad4 plays a pivotal role in TFG-β/Smad signaling pathway, regulating cell proliferation, differentiation, and apoptosis. Knockdown of Smad4 results in loss of a tumor-suppressive function of TFG-β only, *i.e.*, cell cycle arrest, but has no effect on EMT induced by TGF-β in concert with the Ras/Erk pathway [23]. Loss of Smad4 might underlie the functional shift of TGF-β from a tumor suppressor to a tumor promoter [24].

The protein level of Smad4 in lymph node metastases [25] and liver metastases [26] of CRC was significantly lower than in primary tumors. Suppressing Smad4 may enhance the proliferation, migration and invasion of the HCT116 cell line [27]. Papageougis *et al*. [28] found that loss of function of Smad4 and retention of intact TGF-β receptors could synergistically increase the levels of VEGF, enhanced migration of CRC cells with a corresponding increase in matrix metalloprotease-9 enhanced hypoxia-induced GLUT1 expression, increased aerobic glycolysis, and resistance to 5′-fluoruracil-mediated apoptosis.

#### 2.2.4. Nonmetastatic Protein 23

*Nonmetastatic protein 23* (NM23) gene was isolated as a putative metastatic suppressor gene. NM23-H1 is one isotype of the human *NM23* gene. Over-expression of NM23-H1 in metastatic cell lines reduced cell motility *in vitro* assays and metastatic potential in xenograft models [29]. Melanoma and breast cancer with a low expression of NM23 appeared to be more at risk of developing brain metastases [30,31]. Suzuki *et al*. [30] found that NM23-H1 strongly inhibited the liver metastasis of HT-29 cells in nude mice and inhibited the epidermal growth factor-induced cell migration *in vitro*. NM23-H1 expression negatively correlated with intratumoral MVD [32], suggesting NM23 may have a tumor suppressive effect by inhibiting neoplastic angiogenesis. According to the result of a tissue microarray with 130 CRC patients, NM23 expression was higher in the cancer tissue than in adjacent non-neoplastic mucosa, and patients with higher NM23 protein intensity turned out to have a longer disease-free survival [33]. However, no significant difference was found between primary and metastatic CRC tissue [34], which implies that increased NM23 maybe important only in the early stage of CRC.

#### 2.2.5. T-cell Lymphoma Invasion and Metastasis-Inducing Protein 1

TIAM1 is expressed in almost all adult tissues, with especially high expression in the brain and testis [35]. It is a guanine nucleotide exchange factor that activates *Rac* and *Cdc42*[36,37]. Increased TIAM1 expression is associated with increased metastatic potential in colon cancer cell lines [38,39]. Evidence also implicated TIAM1 as a crucial component of the PAR complex in regulating neuronal (axonal) and epithelial (apical-basal) polarity [40].

#### 2.2.6. S100A4

S100A4 is known to be capable of modulating intercellular adhesion and invasive and metastatic properties of cancer cells [41]. High S100A4 expression was associated with tumor stage and secondary metastasis of CRC, presenting with a prognostic effect for disease recurrence and survival [42,43]. Furthermore, S100A4 reduces the expression of occludin, and stimulates p53 expression in brain microvascular endothelial cells, so as to disturb the normal construction of the blood-brain barrier, indicating its potential role in the formation of brain metastases [44].

#### 2.2.7. Src

Irby *et al*. [45] first demonstrate *Src* is an oncogene in CRC. Sequencing of genome in a large set of tumor biopsies confirmed the expression of *Src* mutant in about 1% of the CRC analyzed, which means that *Src* oncogenic mutations are a rare event in CRC. Despite the rare incidence of *Src*, its activity is an independent indicator of poor clinical prognosis in CRC [46,47]. High-level expression of *Src* in primary CRC is predictive for tumor recurrence and metastasis formation [48]. Antibodies targeting Src family kinases such like bosutinib, dasatinib, and saracatinib can strongly impact the migration, invasion, and angiogenesis of CRC cells [49–52].

## 3. The Role of the Blood-Brain Barrier in Brain Metastasis

The blood-brain barrier (BBB) is formed by a complex system of endothelial cells, astroglia, pericytes, with continuous tight junctions that restrict the passage of most circulating cells, bioactive molecules, and therapeutics [53,54]. A physical blocking effect takes place. In addition, an electrically-selective effect is experienced, by which, due to bioactive membrane-proteins on the surfaces the BBB, only the entry of agents with low molecular weights (diameter less than 20 nm) is allowed through passive diffusion, or in most situations, by bioactive memgrane-transporters on the surfaces of brain capillary endothelias and astrocytic endfeet, such as multidrug resistance proteins (MRPs), organic anion transporting polypeptides (OATPs) or P-glycoprotein (Pgp) [55–59].

The tight junctions between endothelials become “loose” when under the burden of primary or metastatic brain tumors, resulting in a high permeability, which allows circulating tumor cells enter the brain. However, in spite of increasing chances for tumor cells to enter into the brain parenchyma, most systemic chemotherapeutic agents are still too large to cross the BBB. Therefore, the brain becomes a “refrigerator” for metastatic tumor cells and poorly-responsed to conventional chemotherapies and biotherapies [58].

The molecular mechanisms regarding tumor cells crossing the BBB have not yet been completely clarified. The related evidence has mostly been based on researche about Brain metastases of breast cancer. Evidence showed that CXCR4, a receptor of chemokine CXCL12, can induce blood vessel instability and increase the permeability of brain endotheials. Inhibition of the pathway of CXCR4/CXCL12 would decrease breast cancer cells migration as well as vascular permeability [60]. Colorectal carcinoma cells also express a higher level of CXCR4 than normal intestinal epithelias. Immunohistochemical analysis confirmed strong expression of CXCR4 in all brain metastases sampled [5] as well as liver and lymph node metastases [61,62], indicating the role of CXCR4/CXCL12 pathway may also induce the process.

On the other hand, vascular endothelial growth factor (VEGF), an important predictive factor for various malignancies including CRC, was also found to increase brain microvascular endothelial cell (BMEC) monolayer permeability by modulating transendothelial migration [63], reducing occludin expression and disrupting ZO-1 and occludin organization, and to lead to tight junction disassembly [64,65].

## 4. Brain Microenvironment and Tumor Metastasis

The brain extracellular matrix is lack of fibronectin and collagen, which is common in other systemic organs, but full of tenascin, laminin and glycosaminoglycans like heparan sulfate and hyaluronic acid [66]. The bio-function of various kinds of glial cells are not alike interstitial cells from other organs. As a result, the tumor microenvironment in brain tissue is quite distinct from other metastatic target organs such as liver and lung.

### 4.1. Extracellular Matrix

#### 4.1.1. Matrix Metalloproteinases

Matrix metalloproteinases (MMPs) are zinc endopeptidases that degrade the extracellular matrix proteins. By remodeling connective tissue, *i.e.*, degradating type I collagen, laminin and fibronectin, MMPs can assist tumor cells to pass through the extracellular matrix and enhance the migration of tumor cells [67,68]. They also participate in the process of epithelial-mesenchymal transition during tumor development [69]. Overexpression of MMP-1, -2, -3, -7, -9, -13 has been demonstrated in human CRC, correlating with a late-stage of diseases and poor prognosis. Each substrate plays its relatively-specific role in microsatellite instability and malignant transformation in CRC. In addition, each substrate plays its relatively-specific role in microsatellite instability and malignant transformation [69]. MMPs were found to be over-expressed in brain metastases compared with their primary tumors [70,71]. The development of experimental brain metastasis was significantly decreased by selective MMP inhibitors [71].

#### 4.1.2. Heparanase

Heparanase is the only endogenous glucuronidase found in mammalian cells up to now, and can degrade the heparan sulfate which is the side chain of heparin sulfate proteoglycans existing in the extracellular matrix and basement membrane, also known to destroy the blood-brain barrier [72]. Heparanase mediates the expression and subcellular localization of guanine nucleotide exchange factor-H1 (GEF-H1), a component of a syndecan signaling complex, thus mediates the cross-talk between tumor cells and brain endothelia and regulates the cytoskeletal dynamics of brain metastatic melanoma and breast cancer cells [73,74]. The expression level of heparanase is a significant independent risk factor for hematogenous metastasis in CRC [75].

### 4.2. Glial Cells

#### 4.2.1. Astrocyte

Astrocytes, also known as astroglia, are the most abundant cell of the human brain, performing bioeffects including composing the blood-brain barrier, provision of nutrients to the neurons, maintenance of extracellular ion balance, and damage repair. When co-cultured with astrocytes, lung adenocarcinoma cells and breast cancer cells presented with a significantly higher growth rates [76]. Such factors suggested that the astrocytes do play a vital role in the progressions of brain tumors. Lin *et al*. [77] demonstrated that astrocytes protect tumor cells from chemotherapy by sequestering intracellular calcium through gap junction communication between astrocytes and tumor cells. Moreover, through paracrine signaling, astrocytes upregulate extracellular matrix compositions with a pro-neoplastic effect such as MMP and heparanase [78,79].

#### 4.2.2. Microglia

Microglia acts as the main form of active immune defense in brain, serving as the resident macrophages of the central nerve system. Generally, microglia accumulate around tumor cells and *in vitro* conditioned medium from primary cultured mouse microglia which inhibits the proliferation of tumor cells [80]. Nitric oxide (NO) mediates the tumoricidal effect of microglia [81], however, brain-metastatic CRC cells may also have a protective mechanism inhibiting NO production [82].

### 4.3. Blood Supply for Brain Metastases

It is necessary for circulating tumor cells to form a sustained blood supply for continued tumor growth in the new metastatic site. In fact, metastatic brain tumor cells located less than 100 μm from a blood vessel are viable. The onset of angiogenesis is activated by the combined effects of proangiogenetic and antiangiogenetic molecules [83]. Vascular endothelial growth factor (VEGF) is the most important bioactive molecule to promote the neoangiogenesis by stimulating the proliferation and migration of endothelial cells. In an experiment on mice models of brain metastasis from colon cancer, the expression of *VEGF* mRNA and protein in the metastatic tumors were observed, which was correlated with angiogenesis and growth of brain metastasis. This pro-angiogenesis effect could be inhibited by transfecting with the *antisense-BEGF165* gene [84]. The result indicated that VEGF is necessary for production and growth of brain metastasis.

Many studies suggest that the microvessel density (MVD) within the neoplasms correlates with the aggressiveness of the disease. A significantly higher MVD was observed in CRC with liver metastatic disease compared with the tumors without liver metastasis [85]. This generalization, however, does not extend to brain metastases. In experimental brain metastasis from colon cancer, the MVD within these lesions was lower than the MVD in the surrounding uninvolved brain. The metastases contained large blood vessels with dilated lumens, which are thought to be a form of vascular remodeling by nonsprouting angiogenesis [83,84].

## 5. Site-Specific Metastatic Factor

### 5.1. Chemokine

Chemokines is a family of small proteins secreted by cells (8–10 kilodaltons in size), act as a chemoattractant to guide the migration of the chemokines receptor-expressing cells like immune bells and various tumor cells, and participate in early development, angiogenesis and lymphogenesis, tumor growth and metastases [86]. Chemokine(C-X-C motif) ligand 12 (CXCL12) is released in high amounts by certain organ, such as liver, lung, bone, as well as brain parenchyma. CXCR4, the specific receptor of CXCL12, is highly-expressed in cancer cells compared with homological normal tissue [86–88]. These tumor cells may metastasize to organs that secret CXCL12. The attraction effect between CXCL12 and CXCR4 causes breast cancer cells and non-small cell lung cancer cells to migrate into brain, where cancer cells proliferate and form metastatic tumors [60,87,89]. CXCL12 can significantly increase the number of clones in CRC cell lines *in vitro*[61]. The expression of CXCRL12 and nuclear CXCR4 predicts lymph node metastases and liver metastases in CRC [61,62,90]. Mangan *et al*. [55] first confirmed the association between CXCR4 and brain metastases in CRC. Immunohistochemical staining was performed on tumor specimens in 11 patients who underwent resection of brain metastases. All of the specimens were strongly positive for CXCR4, with a primarily nuclear location of CXCR4 expression, combined with high pulmonary and low hepatic metastases. Furthermore, CXCL12 increases VEGF expression and cell proliferation; the expression of CXCR4 and VEGF is correlated [61,88].

### 5.2. MicroRNA

MicroRNAs (miRNAs) are a large family of small non-coding RNAs that negatively control gene expression at the mRNA and protein levels. Evidence indicates that miRNAs present with a tissue-specific expression profile in cancer and adjacent non-tumorous tissue [91,92], and may be key players in the regulation of tumor cell invasion and metastasis. Researchers have located several miRNAs associated with brain metastasis of melanoma and lung cancer including miR-145 and miR-328 [93–95]. In our previous study, we also found 2 miRNAs were down-regulated and 17 miRNAs were up-regulated in the brain metastatic colorectal carcinomas [96]. These miRNAs may hold great potential as targets for histology-specific diagnosis and treatment.

## 6. Models for Metastatic Brain Tumors

A stable animal model, mimicing the process of metastasis, is essential for further understanding the pathogenesis and treatment of brain metastasis. There are various methods of animal models for studying brain tumors (see in Table 2). Norihiko *et al*. [97] believed that different methods of animal models reflect different pathological processes because of the unique composition of the brain. In the stereotactic intracerebral injection model, the tumor proliferation at the injection site and the infiltration into the brain parenchyma were observed. The intrathecal (cisterna magna) injection model reflects leptomeningeal carcinomatosis, in which metastasis to the meninges was observed. Last but not least, in the intracarotid injection model, both perivascular and invasive proliferations were ovserved, as a result of which this model reflects most objectively the process of haematogenous metastasis.

However, until now, there is no spontaneous brain metastasis models of CRC ever reported. The most commonly-used models for studying Brain metastases are experimental metastatic models, namely the tumor cells are directly inoculated into circulation and colonize in the brain, thereby resembling only the last steps of metastasis: survival in the circulation, extravasation, and colonization in the target organs. Rashidi *et al*. recently established a spontaneous Brain metastases model of lung cancer by surgical orthotopic implantation (SOI) techenique, thus making it possible to mimic the initial process of malignancy metastasis.

## 7. Conclusions

The incidence of brain metastasis from CRC is increasing as the improvement of systemic disease is achieved. The severe neurological disability of brain metastasis followed by poor prognosis and miserable quality of life makes it more and more urgent to further investigate the process of brain metastasis from CRC. However, due to a relatively low incidence, little is known so far about the underlying mechanisms of brain metastasis from CRC. Certain molecule candidates have been found to be related to CRC brain metastasis. A stable *in vivo* model of CRC brain metastasis is necessary for further understanding of the underlying mechanisms so as to propose a new strategy for CRC patients with brain metastasis and improve their life quality.

## Figures and Tables

**Table 1:** Genes associated with metastatic potential of colorectal cancer.

OMIM No.	Gene	Chromosome Location	Function(s) of Protein
107269	*CD44*	11pter-p13	An integral cell membrane glycoprotein with a postulated role in matrix adhesion lymphocyte activation and lymph node homing
114210	*S100A4*	1q21.3	Increases endothelial cell motility, increases invasive properties through deregulation of the extracellular matrix
120361	*MMP-9*	20q13.12	Extracellular matrix degradation
156490	*NM23*	17q21.3	A histidine kinase related to cell proliferative activity by phosphorylating KSR and leading to decreased ERK1/2 activation
190070	*KRAS*	12p12.1	Encode GDP/GTP-binding proteins involved in signal transduction in cellular proliferation, differentiation, and senescence
190090	*SRC*	20q11.23	Regulating cell division, motility, adhesion, angiogenesis, and survival
192240	*VEGF*	6p21.1	Angiogenic growth factor, controlling blood vessel formation and permeability
600687	*TIAM1*	21q22.11	Rac-specific guanine nucleotide exchange factor, directly mediate Ras activation of Rac.
600993	*SMAD4*	18q21.2	Transcription factor, pivotal role in signal transduction of TGF-β
	*RHOGDI2*	11p11.2	Regulates function of Rho and Rac, involved in cell signaling, proliferation, cytoskeletal organization, and secretion

**Table 2:** Methods of animal models for brain tumors.

Authors	Methods of Modeling	Tumor Cell Lines	Animals	Primary Tumors
Bullard, D.E. [98]	Intracerebral injection	D-54 MG, U-118 MG, U-251 MG	nu/nu genotype, NIH Swiss, BALB/c	Gliomatosis
Yoshida, T. [99]	Intracisternal inoculation	C6, 9L glioma	Wistar, Fischer 344 rats	Meningeal gliomatosis
Schackert, G. [100]	Intracarotid injection	K-1735, B16 melanoma UV-2237 fibrosarcoma	C3H/HeN, C57BL/6, C57BL/6 × C3H/HeN F1	Melanoma and fibrosarcoma
Kim, L.S. [101]	Intracarotid injection	MDA-MB-231	NCr–nu/nu mice	Breast cancer
Mendes, O. [102]	Left ventricular injection	ENU 1564	BD-IV rats	Breast cancer
Zhang, Z. [103], Saito, N. [104]	Intracarotid injection	Lewis lung carcinoma cells	C57BL/6NCrj mice	Lung cancer
Rashidi, B. [105]	SOI (right lung)	Lewis lung carcinoma cells	BALB/c mice	Lung cancer
Yano, S. [84]	Intracarotid injection	Colon carcinoma KM12SM	NCr–nu/nu mice	Colon carcinoma
Weilemann, F. [106]	Intracarotid injection	CT-26	BALB/c mice	Colon carcinoma
Wang, J. [107]	Intracerebral implantation	pts brain metastaes	rnu/rnu Rowett rats	Ovary, colon, lung cancer, and melanoma

## References

[1] Jemal A., Bray F., Center M.M., Ferlay J., Ward E., Forman D. (2011). Global Cancer Statistics. CA: Cancer J. Clin.

[2] Kruser T.J., Chao S.T., Elson P., Barnett G.H., Vogelbaum M.A., Angelov L., Weil R.J., Pelley R., Suh J.H. (2008). Multidisciplinary management of colorectal brain metastases—A retrospective study. Cancer.

[3] Rahmathulla G., Toms S.A., Weil R.J. (2012). The molecular biology of brain metastasis. J. Oncol..

[4] Cohen S.J., Wang H., Rogatko A., Meropol N.J., Sundermeyer M.L. (2005). Changing Patterns of Bone and Brain Metastases in Patients with Colorectal Cancer. Clin. Colorectal Cancer.

[5] Mongan J.P., Fadul C.E., Cole B.F., Zaki B.I., Suriawinata A.A., Ripple G.H., Tosteson T.D., Pipas J.M. (2009). Brain metastases from colorectal cancer: Risk factors, incidence, and the possible role of chemokines. Clin. Colorectal Cancer.

[6] Jiang X.-B., Yang Q.-Y., Sai K., Zhang X.-H., Chen Z.-P., Mou Y.-G. (2011). Brain metastases from colorectal carcinoma: A description of 60 cases in a single Chinese cancer center. Tumor Biol.

[7] Jung M., Ahn J.B., Chang J.H., Suh C.O., Hong S., Roh J.K., Shin S.J., Rha S.Y. (2011). Brain metastases from colorectal carcinoma: Prognostic factors and outcome. J. Neuro-Oncol.

[8] Farnell G.F., Buckner J.C., Cascino T.L., Oconnell M.J., Schomberg P.J., Suman V. (1996). Brain metastases from colorectal carcinoma—The long term survivors. Cancer.

[9] Lin B.-R., Chang T.-C., Lee Y.-C., Lee P.-H., Chang K.-J., Liang J.-T. (2009). Pulmonary Resection for Colorectal Cancer Metastases: Duration Between Cancer Onset and Lung Metastasis as an Important Prognostic Factor. Ann. Surg. Oncol.

[10] Franco-Hernandez C., Martinez-Glez V., Arjona D., de Campos J.M., Isla A., Gutierrez M., Vaquero J., Rey J.A. (2007). EGFR sequence variations and real-time quantitative polymerase chain reaction analysis of gene dosage in brain metastases of solid tumors. Cancer Genet. Cytogenet.

[11] Scartozzi M., Bearzi I., Berardi R., Mandolesi A., Fabris G., Cascinu S. (2004). Epidermal growth factor receptor (EGFR) status in primary colorectal tumors does not correlate with EGFR expression in related metastatic sites: Implications for treatment with EGFR-targeted monoclonal antibodies. J. Clin. Oncol.

[12] Barault L., Veyrie N., Jooste V., Lecorre D., Chapusot C., Ferraz J.-M., Lievre A., Cortet M., Bouvier A.-M., Rat P. (2008). Mutations in the RAS-MAPK, PI(3)K (phosphatidylinositol-3-OH kinase) signaling network correlate with poor survival in a population-based series of colon cancers. Int. J. Cancer.

[13] Tie J., Lipton L., Desai J., Gibbs P., Jorissen R.N., Christie M., Drummond K.J., Thomson B.N.J., Usatoff V., Evans P.M. (2011). KRAS Mutation Is Associated with Lung Metastasis in Patients with Curatively Resected Colorectal Cancer. Clin. Cancer Res.

[14] Resnick D.K., Resnick N.M., Welch W.C., Cooper D.L. (1999). Differential expressions of CD44 variants in tumors affecting the central nervous system. Molecular Diagn.

[15] Bendardaf R., Algars A., Elzagheid A., Korkeila E., Ristamaki R., Lamlum H., Collan Y., Syrjanen K., Pyrhonen S. (2006). Comparison of CD44 expression in primary tumours and metastases of colorectal cancer. Oncol. Rep.

[16] Zaytseva Y.Y., Rychahou P.G., Gulhati P., Elliott V.A., Mustain W.C., O’Connor K., Morris A.J., Sunkara M., Weiss H.L., Lee E.Y. (2012). Inhibition of Fatty Acid Synthase Attenuates CD44-Associated Signaling and Reduces Metastasis in Colorectal Cancer. Cancer Res.

[17] Seraj M.J., Harding M.A., Gildea J.J., Welch D.R., Theodorescu D. (2000). The relationship of BRMS1 and RhoGDI2 gene expression to metastatic potential in lineage related human bladder cancer cell lines. Clin. Exp. Metastasis.

[18] Harding M.A., Theodorescu D. (2007). RhoGDI2: A new metastasis suppressor gene: Discovery and clinical translation. Urol. Oncol.

[19] Agarwal N.K., Chen C.H., Cho H., Boulbès D.R., Spooner E., Sarbassov D.D. (2012). Rictor regulates cell migration by suppressing RhoGDI2. Oncogene.

[20] Li X., Wang J., Zhang X., Zeng Y., Liang L., Ding Y. (2012). Overexpression of RhoGDI2 correlates with tumor progression and poor prognosis in colorectal carcinoma. Ann. Surg. Oncol.

[21] Fujita A., Shida A., Fujioka S., Kurihara H., Okamoto T., Yanaga K. (2012). Clinical significance of Rho GDP dissociation inhibitor 2 in colorectal carcinoma. Int. J. Clin. Oncol.

[22] Li J.-F., Zheng Z., Yu B.-Q., Qu Y., Zhu Z.-G., Liu B.-Y. (2012). Screening and identification of genes associated with multi-drug resistance in colonic cancer. Zhonghua Wei Chang Wai Ke Za Zhi.

[23] Levy L., Hill C.S. (2005). Smad4 dependency defines two classes of transforming growth factor {beta} (TGF-{beta}) target genes and distinguishes TGF-{beta}-induced epithelial-mesenchymal transition from its antiproliferative and migratory responses. Mol. Cell Biol.

[24] Zhang B., Halder S.K., Kashikar N.D., Cho Y.J., Datta A., Gorden D.L., Datta P.K. (2010). Antimetastatic role of Smad4 signaling in colorectal cancer. Gastroenterology.

[25] Alhopuro P., Alazzouzi H., Sammalkorpi H., Davalos V., Salovaara R., Hemminki A., Jarvinen H., Mecklin J.P., Schwartz S., Aaltonen L.A. (2005). SMAD4 levels and response to 5-fluorouracil in colorectal cancer. Clin. Cancer Res..

[26] Losi L., Bouzourene H., Benhattar J. (2007). Loss of Smad4 expression predicts liver metastasis in human colorectal cancer. Oncol. Rep.

[27] Shi Q., Zhong Y.S., Yao L.Q., Li Q.L., Ren Z., Liu X.P., Shi F.M. (2012). Down-regulation of Smad4 enhances proliferation and invasion of colorectal carcinoma HCT116 cells and up-regulates Id2. Mol. Med. Rep.

[28] Papageorgis P., Cheng K., Ozturk S., Gong Y., Lambert A.W., Abdolmaleky H.M., Zhou J.R., Thiagalingam S. (2011). Smad4 inactivation promotes malignancy and drug resistance of colon cancer. Cancer Res.

[29] Marino N., Marshall J.C., Steeg P.S. (2011). Protein-protein interactions: A mechanism regulating the anti-metastatic properties of Nm23-H1. Naunyn. Schmiedebergs Arch. Pharmacol.

[30] Suzuki E., Ota T., Tsukuda K., Okita A., Matsuoka K., Murakami M., Doihara H., Shimizu N. (2004). *nm23-H1* reduces *in vitro* cell migration and the liver metastatic potential of colon cancer cells by regulating myosin light chain phosphorylation. Int. J. Cancer.

[31] Stark A.M., Tongers K., Maass N., Mehdorn H.M., Held-Feindt J. (2005). Reduced metastasis-suppressor gene mRNA-expression in breast cancer brain metastases. J. Cancer Res. Clin. Oncol.

[32] Elagoz S., Egilmez R., Koyuncu A., Muslehiddinoglu A., Arici S. (2006). The intratumoral microvessel density and expression of bFGF and nm23-H1 in colorectal cancer. Pathol. Oncol. Res.

[33] Oliveira L.A., Artigiani-Neto R., Waisberg D.R., Fernandes L.C., de Lima F.O., Waisberg J. (2010). NM23 protein expression in colorectal carcinoma using TMA (tissue microarray): Association with metastases and survival. Arq. Gastroenterol..

[34] Sarris M., Lee C.S. (2001). nm23 protein expression in colorectal carcinoma metastasis in regional lymph nodes and the liver. Eur. J. Surg. Oncol.

[35] Habets G.G.M., Vanderkammen R.A., Stam J.C., Michiels F., Collard J.G. (1995). Sequence of human invasion-inducing TIAM1 gene, its conservation in evolution and its expression in tumor-cell lines of lines of different tissue origin. Oncogene.

[36] Ehler E., van Leeuwen F., ollard J.G., Salinas P.C. (1997). Expression ofTiam-1in the Developing Brain Suggests a Role for the Tiam-1-Rac Signaling Pathway in Cell Migration and Neurite Outgrowth. Mol. Cell Neurosci.

[37] Minard M.E., Ellis L.M., Gallick G.E. (2006). Tiam1 regulates cell adhesion, migration and apoptosis in colon tumor cells. Clin. Exp. Metastasis.

[38] Minard M.E., Herynk M.H., Collard J.G., Gallick G.E. (2005). The guanine nucleotide exchange factor Tiam1 increases colon carcinoma growth at metastatic sites in an orthotopic nude mouse model. Oncogene.

[39] Liu L., Wu D.-H., Ding Y.-Q. (2005). Tiam1 gene expression and its significance in colorectal carcinoma. World J. Gastroenterol.

[40] Mertens A.E., Pegtel D.M., Collard J.G. (2006). Tiam1 takes PARt in cell polarity. Trends Cell Biol.

[41] Sherbet G.V., Lakshmi M.S. (1998). S100A4 (MTS1) calcium binding protein in cancer growth, invasion and metastasis. Anticancer Res.

[42] Gongoll S., Peters G., Mengel M., Piso P., Klempnauer J., Kreipe H., Von Wasielewski R. (2002). Prognostic significance of calcium-binding protein S100A4 in colorectal cancer. Gastroenterol.

[43] Kwak J.M., Lee H.J., Kim S.H., Kim H.K., Mok Y.J., Park Y.T., Choi J.S., Moon H.Y. (2010). Expression of protein S100A4 is a predictor of recurrence in colorectal cancer. World J. Gastroenterol.

[44] Nishioku T., Furusho K., Tomita A., Ohishi H., Dohgu S., Shuto H., Yamauchi A., Kataoka Y. (2011). Potential role for S100A4 in the disruption of the blood-brain barrier in collagen-induced arthritic mice, and animal model of rheumatoid arthritis. Neuroscience.

[45] Irby R.B., Mao W.G., Coppola D., Kang J., Loubeau J.M., Trudeau W., Karl R., Fujita D.J., Jove R., Yeatman T.J. (1999). Activating SRC mutation in a subset of advanced human colon cancers. Nat. Genet.

[46] Aligayer H., Boyd D.D., Heiss M.M., Abdalla E.K., Curley S.A., Gallick G.E. (2002). Activation of Src kinase in primary colorectal carcinoma: An indicator of poor clinical prognosis. Cancer.

[47] Maurer G.D., Leupold J.H., Schewe D.M., Biller T., Kates R.E., Hornung H.-M., Lau-Werner U., Post S., Allgayer H. (2007). Analysis of specific transcriptional regulators as early predictors of independent prognostic relevance in resected colorectal cancer. Clin. Cancer Res.

[48] de Heer P., Koudijs M.M., van de Velde C.J.H., Aalbers R.I.J.M., Tollenaar R.A.E.M., Putter H., Morreau J., van de Water B., Kuppen P.J.K. (2008). Combined expression of the non-receptor protein tyrosine kinases FAK and Src in primary colorectal cancer is associated with tumor recurrence and metastasis formation. Eur. J. Surg. Oncol.

[49] Sirvent A., Benistant C., Pannequin J., Veracini L., Simon V., Bourgaux J.F., Hollande F., Cruzalegui F., Roche S. (2010). Src family tyrosine kinases-driven colon cancer cell invasion is induced by Csk membrane delocalization. Oncogene.

[50] Nam J.S., Ino Y., Sakamoto M., Hirohashi S. (2002). Src family kinase inhibitor PP2 restores the E-cadherin/catenin cell adhesion system in human cancer cells and reduces cancer metastasis. Clin. Cancer Res.

[51] Boyd D.D., Wang H., Avila H., Parikh N.U., Kessler H., Magdolen V., Gallick G.E. (2004). Combination of an src kinase inhibitor with a novel pharmacological antagonist of the urokinase receptor diminishes *in vitro* colon cancer invasiveness. Clin. Cancer Res.

[52] Kopetz S., Lesslie D.P., Dallas N.A., Park S.I., Johnson M., Parikh N.U., Kim M.P., Abbruzzese J.L., Ellis L.M., Chandra J., Gallick G.E. (2009). Synergistic Activity of the Src Family Kinase Inhibitor Dasatinib and Oxaliplatin in Colon Carcinoma Cells Is Mediated by Oxidative Stress. Cancer Res.

[53] Petty M.A., Lo E.H. (2002). Junctional complexes of the blood-brain barrier: Permeability changes in neuroinflammation. Prog. Neurobiol.

[54] Meijer O.C., de Lange E.C.M., Breimer D.D., de Boer A.G., Workel J.O., de Kloet E.R. (1998). Penetration of dexamethasone into brain glucocorticoid targets is enhanced in mdr1A P-glycoprotein knockout mice. Endocrinology.

[55] Loscher W., Potschka H. (2005). Role of drug efflux transporters in the brain for drug disposition and treatment of brain diseases. Prog. Neurobiol.

[56] Butt A.M., Jones H.C., Abbott N.J. (1990). Electrical-resistance across the blood-brain-barrier in anesthetized rats—a developmental study. J. Physiol. (London).

[57] Deli M.A., Abraham C.S., Kataoka Y., Niwa M. (2005). Permeability studies on *in vitro in vitro* blood-brain barrier models: Physiology, pathology, and pharmacology. Cell Mol. Neurobiol.

[58] Bart J., Groen H.J.M., Hendrikse N.H., van der Graaf W.T.A., Vaalburg W., de Vries E.G.E. (2000). The blood-brain barrier and oncology: New insights into function and modulation. Cancer Treat. Rev.

[59] Harhaj N.S., Antonetti D.A. (2004). Regulation of tight junctions and loss of barrier function in pathophysiology. Int. J. Biochem. Cell Biol.

[60] Lee B.C., Lee T.H., Avraham S., Avraham H.K. (2004). Involvement of the chemokine receptor CXCR4 and its ligand stromal cell-derived factor 1 alpha in breast cancer cell migration through human brain microvascular endothelial cells. Mol. Cancer Res.

[61] Ottaiano A., Franco R., Talamanca A.A., Liguori G., Tatangelo F., Delrio P., Nasti G., Barletta E., Facchini G., Daniele B. (2006). Overexpression of both CXC chemokine receptor 4 and vascular endothelial growth factor proteins predicts early distant relapse in stage II–III colorectal cancer patients. Clin. Cancer Res.

[62] Yoshitake N., Fukui H., Yamagishi H., Sekikawa A., Fujii S., Tomita S., Ichikawa K., Imura J., Hiraishi H., Fujimori T. (2008). Expression of SDF-1 alpha and nuclear CXCR4 predicts lymph node metastasis in colorectal cancer. Br. J. Cancer.

[63] Lee T.H., Avraham H.K., Jiang S.X., Avraham S. (2003). Vascular endothelial growth factor modulates the transendothelial migration of MDA-MB-231 breast cancer cells through regulation of brain microvascular endothelial cell permeability. J. Biol. Chem.

[64] Wang W., Dentler W.L., Borchardt R.T. (2001). VEGF increases BMEC monolayer permeability by affecting occludin expression and tight junction assembly. Am. J. Physiol.-Heart Circul. Physiol.

[65] Harhaj N.S., Felinski E.A., Wolpert E.B., Sundstrom J.M., Gardner T.W., Antonetti D.A. (2006). VEGF activation of protein kinase C stimulates occludin phosphorylation and contributes to endothelial permeability. Invest. Ophthalmol. Vis. Sci.

[66] Bonneh-Barkay D., Wiley C.A. (2009). Brain Extracellular Matrix in Neurodegeneration. Brain Pathol.

[67] Hotary K., Li X.-Y., Allen E., Stevens S.L., Weiss S.J. (2006). A cancer cell metalloprotease triad regulates the basement membrane transmigration program. Genes Dev.

[68] Mook O.R.F., Frederiks W.M., Van Noorden C.J.F. (2004). The role of gelatinases in colorectal cancer progression and metastasis. Biochim. Biophys. Acta-Rev. Cancer.

[69] Zucker S., Vacirca J. (2004). Role of matrix metalloproteinases (MMPs) in colorectal cancer. Cancer Metastasis Rev.

[70] Arnold S.M., Young A.B., Munn R.K., Patchell R.A., Nanayakkara N., Markesbery W.R. (1999). Expression of p53, bcl-2, E-cadherin, matrix metalloproteinase-9, and tissue inhibitor of metalloproteinases-1 in paired primary tumors and brain metastasis. Clin. Cancer Res.

[71] Xie T.X., Huang F.J., Aldape K.D., Kang S.H., Liu A.G., Gershenwald J.E., Xie K.P., Sawaya R., Huang S.Y. (2006). Activation of Stat3 in human melanoma promotes brain metastasis. Cancer Res.

[72] Nathoo N., Chahlavi A., Barnett G.H., Toms S.A. (2005). Pathobiology of brain metastases. J. Clin. Pathol.

[73] Ridgway L.D., Wetzel M.D., Marchetti D. (2010). Modulation of GEF-H1 Induced Signaling by Heparanase in Brain Metastatic Melanoma Cells. J. Cell Biochem.

[74] Ridgway L.D., Wetzel M.D., Ngo J.A., Erdreich-Epstein A., Marchetti D. (2012). Heparanase-induced GEF-H1 signaling regulates the cytoskeletal dynamics of brain metastatic breast cancer cells. Mol. Cancer Res.

[75] Sato T., Yamaguchi A., Goi T., Hirono Y., Takeuchi K., Katayama K., Matsukawa S. (2004). Heparanase expression in human colorectal cancer and its relationship to tumor angiogenesis, hernatogenous metastasis, and prognosis. J. Surg. Oncol.

[76] Langley R.R., Fan D., Guo L.X., Zhang C.Y., Lin Q.T., Brantley E.C., McCarty J.H., Fidler I.J. (2009). Generation of an immortalized astrocyte cell line from H-2K(b)-tsA58 mice to study the role of astrocytes in brain metastasis. Int. J. Oncol.

[77] Lin Q.T., Balasubramanian K., Fan D., Kim S.J., Guo L.X., Wang H., Bar-Eli M., Aldape K.D., Fidler I.J. (2010). Reactive Astrocytes Protect Melanoma Cells from Chemotherapy by Sequestering Intracellular Calcium through Gap Junction Communication Channels. Neoplasia.

[78] Marchetti D., Li J., Shen R. (2000). Astrocytes contribute to the brain-metastatic specificity of melanoma cells by producing heparanase. Cancer Res.

[79] Mendes O., Kim H.T., Lungu G., Stoica G. (2007). MMP2 role in breast cancer brain metastasis development and its regulation by TIMP2 and ERK1/2. Clin. Exp. Metastasis.

[80] Noda M., Seike T., Fujita K., Yamakawa Y., Kido M., Iguchi H. (2009). The role of immune cells in brain metastasis of lung cancer cells and neuron-tumor cell interaction. Ross. Fiziol. Zh. Im. I. M. Sechenova.

[81] Murata J., RicciardiCastagnoli P., Mange P.D.L., Martin F., JuilleratJeanneret L. (1997). Microglial cells induce cytotoxic effects toward colon carcinoma cells: Measurement of tumor cytotoxicity with a gamma-glutamyl transpeptidase assay. Int. J. Cancer.

[82] Murata J.I., Corradin S.B., Janzer R.C., Juilleratjeanneret L. (1994). Tumor-cells suppress cytokine-induced nitric-oside (NO) production in cerebral endothelial-cells. Int. J. Cancer.

[83] Fidler I.J., Yano S., Zhang R.D., Fujimaki T., Bucana C.D. (2002). The seed and soil hypothesis: vascularisation and brain metastases. Lancet Oncol.

[84] Yano S., Shinohara H., Herbst R.S., Kuniyasu H., Bucana C.D., Ellis L.M., Davis D.W., McConkey D.J., Fidler I.J. (2000). Expression of vascular endothelial growth factor is necessary but not sufficient for production and growth of brain metastasis. Cancer Res.

[85] Tomisaki S., Ohno S., Ichiyoshi Y., Kuwano H., Maehara Y., Sugimachi K. (1996). Microvessel quantification and its possible relation with liver metastasis in colorectal cancer. Cancer.

[86] Ben-Baruch A. (2008). Organ selectivity in metastasis: Regulation by chemokines and their receptors. Clin. Exp. Metastasis.

[87] Chen G., Wang Z., Liu X.Y., Liu F.Y. (2011). High-level CXCR4 expression correlates with brain-specific metastasis of non-small cell lung cancer. World J. Surg.

[88] Brand S., Dambacher J., Beigel F., Olszak T., Diebold J., Otte J.M., Goke B., Eichhorst S.T. (2005). CXCR4 and CXCL12 are inversely expressed in colorectal cancer cells and modulate cancer cell migration, invasion and MMP-9 activation. Exp. Cell Res.

[89] Muller A., Homey B., Soto H., Ge N.F., Catron D., Buchanan M.E., McClanahan T., Murphy E., Yuan W., Wagner S.N. (2001). Involvement of chemokine receptors in breast cancer metastasis. Nature.

[90] Matsusue R., Kubo H., Hisamori S., Okoshi K., Takagi H., Hida K., Nakano K., Itami A., Kawada K., Nagayama S., Sakai Y. (2009). Hepatic stellate cells promote liver metastasis of colon cancer cells by the action of SDF-1/CXCR4 axis. Ann. Surg. Oncol.

[91] Lu J., Getz G., Miska E.A., Alvarez-Saavedra E., Lamb J., Peck D., Sweet-Cordero A., Ebet B.L., Mak R.H., Ferrando A.A. (2005). MicroRNA expression profiles classify human cancers. Nature.

[92] Schetter A.J., Leung S.Y., Sohn J.J., Zanetti K.A., Bowman E.D., Yanaihara N., Yuen S.T., Chan T.L., Kwong D.L.W., Au G.K.H. (2008). MicroRNA expression profiles associated with prognosis and therapeutic outcome in colon adenocarcinoma. JAMA.

[93] Lu Y., Govindan R., Wang L., Liu P.Y., Goodgame B., Wen W.D., Sezhiyan A., Pfeifer J., Li Y.F., Hua X. (2012). MicroRNA profiling and prediction of recurrence/relapse-free survival in stage I lung cancer. Carcinogenesis.

[94] Arora S., Ranade A.R., Tran N.L., Nasser S., Sridhar S., Korn R.L., Ross J.T.D., Dhruv H., Foss K.M., Sibenaller Z. (2011). MicroRNA-328 is associated with (non-small) cell lung cancer (NSCLC) brain metastasis and mediates NSCLC migration. Int. J. Cancer.

[95] Gaziel A., Menendez S., Segura M.F., Zakrzewski J., Rose A., Kerbel R.S., Darvishian F., Cohen D., Osman I., Hernando E Identification of miRNAs that contribute to melanoma brain metastasis.

[96] Li Z.Y., Gu X.D., Fang Y.T., Xiang J.B., Chen Z.Y. (2012). microRNA expression profiles in human colorectal cancers with brain metastases. Oncol. Lett.

[97] Saito N., Hatori T., Murata N., Zhang Z.-A., Nonaka H., Aoki K., Iwabuchi S., Ueda M. (2008). Comparison of metastatic brain tumour models using three different methods: The morphological role of the pia mater. Int. J. Exp. Pathol.

[98] Bullard D.E., Schold S.C., Bigner S.H., Bigner D.D. (1981). Growth and chemotherapeutic response in athymic mice of tumors arising from human glioma-derived cell-lines. J. Neuropathol. Exp. Neurol.

[99] Yoshida T., Shimizu K., Ushio Y., Hayakawa T., Arita N., Mogami H. (1986). Development of experimental meningeal glimatosis models in rats. J. Neurosurg.

[100] Schackert G., Fidler I.J. (1988). Site-specific metastasis of mouse melanomas and a fibro-sarcoma in the brain or meninges of syngeneic animals. Cancer Res.

[101] Kim L.S., Huang S.Y., Lu W.X., Lev D.C., Price J.E. (2004). Vascular endothelial growth factor expression promotes the growth of breast cancer brain metastases in nude mice. Clin. Exp. Metastasis.

[102] Mendes O., Kim H.T., Stoica G. (2005). Expression of MMP2, MMP9 and MMP3 in breast cancer brain metastasis in a rat model. Clin. Exp. Metastasis.

[103] Zhang Z., Hatori T., Nonaka H. (2008). An experimental model of brain metastasis of lung carcinoma. Neuropathology.

[104] Saito N., Hatori T., Murata N., Zhang Z.A., Ishikawa F., Nonaka H., Iwabuchi S., Samejima H. (2007). A double three-step theory of brain metastasis in mice: The role of the pia mater and matrix metalloproteinases. Neuropathol. Appl. Neurobiol.

[105] Rashidi B., Yang M., Jiang P., Baranov E., An Z.L., Wang X., Moossa A.R., Hoffman R.M. (2000). A highly metastatic Lewis lung carcinoma orthotopic green fluorescent protein model. Clin. Exp. Metastasis.

[106] Weilemann F., Steinmetz A., Kirsch M., Buttler A., Kunze S., Kuhlisch E., Schackert H.K., Schackert G. (2003). Prevention of brain metastasis formation by local expression of interleukin-4 or hemagglutinin antigen. Zentralbl. Neurochir.

[107] Wang J., Daphu I., Pedersen P.H., Miletic H., Hovland R., Mork S., Bjerkvig R., Tiron C., McCormack E., Micklem D. (2011). A novel brain metastases model developed in immunodeficient rats closely mimics the growth of metastatic brain tumours in patients. Neuropathol. Appl. Neurobiol.

